# Treatment-Induced Neuropathy of Diabetes: Focus on Autonomic Neuropathy

**DOI:** 10.7759/cureus.111651

**Published:** 2026-06-28

**Authors:** Benjamin MacIsaac, Richard MacIsaac, Lai Yin Law

**Affiliations:** 1 Department of General Medicine, St Vincent's Hospital, Melbourne, AUS; 2 Department of Endocrinology and Diabetes, St Vincent's Hospital, Melbourne, AUS; 3 Department of Neurology, St Vincent's Hospital, Melbourne, AUS

**Keywords:** autonomic nervous system disease, autonomic neuropathy, diabetes, diabetic neuropathy, insulin, neuropathy, treatment-induced neuropathy of diabetes, treatment neuropathy

## Abstract

Treatment-induced neuropathy of diabetes (TIND) is characterised by the acute onset of peripheral and autonomic neuropathy following rapid correction of chronic hyperglycaemia. We present a case of TIND presenting with severe, treatment-resistant postural hypotension, with a quantitative sudomotor axon reflex test confirming autonomic small fibre neuropathy. This complication is under-recognised compared to early worsening of diabetic retinopathy or peripheral neuropathy but causes significant patient morbidity. Given the limited treatment options once symptoms develop, prevention is critical. Future research should aim to validate gradual HbA1c reduction as an effective preventative strategy and focus on developing targeted treatment options for TIND, especially its autonomic manifestations.

## Introduction

Acute worsening of diabetic microvascular complications is a recognised but poorly understood phenomenon that occurs in association with rapid improvement in glycaemic control, most notably manifesting as worsening retinopathy in both type 1 and type 2 diabetes mellitus [1]. Treatment-induced neuropathy of diabetes (TIND), historically termed “insulin neuritis,” is an acute, small-fibre neuropathy that occurs following a rapid correction of chronic hyperglycaemia [2]. It is an increasingly recognised complication of intensive glycaemic management and may affect up to 10% of patients referred to tertiary diabetic neuropathy clinics [2]. Symptoms typically develop within one to two months following substantial glycated haemoglobin (HbA1c) reduction and commonly include severe neuropathic pain and autonomic dysfunction [2].

Autonomic dysfunction commonly manifests as orthostatic hypotension (a sustained fall in blood pressure on standing) or gastroparesis (delayed gastric emptying). The exact pathophysiology of TIND remains incompletely understood, with current theories suggesting that abrupt glycaemic shifts trigger maladaptive neurovascular responses, including impaired autoregulation of endoneuronal blood flow, microvascular ischaemia, and neuronal metabolic stress, ultimately leading to acute small-fibre nerve injury [3-5]. Treatment after symptom onset is largely supportive and often provides limited symptomatic relief [2]. Prevention is therefore critical and relies on achieving a gradual, controlled reduction in HbA1c levels [2]. We present a case of TIND in which autonomic neuropathy was the predominant manifestation, an under-recognised yet profoundly debilitating complication. This case also highlights the practical challenges of implementing a graded reduction in HbA1c within routine clinical practice.

## Case presentation

A 51-year-old East Timorese man with type 2 diabetes mellitus presented to the emergency department with recurrent syncope and orthostatic hypotension following a recent rapid correction of chronic hyperglycaemia. He had collapsed at home and reported a three-week history of abrupt-onset syncope, distal paraesthesia, nausea, vomiting, and postprandial gastric fullness. There were no urinary symptoms suggestive of autonomic bladder dysfunction. He had experienced an unintentional weight loss of approximately 35 kg over the preceding six months.

His medical history included type 2 diabetes mellitus, schizophrenia, and untreated bilateral cataracts. Historically, glycaemic control had been poor due to insulin non-adherence related to paranoid beliefs surrounding his diagnosis and treatment. Three months prior to presentation, he was admitted with symptomatic hyperglycaemia and ketosis without acidosis, at which time glycated haemoglobin (HbA1c) was 16.1% (151 mmol/mol). He was commenced on a co-formulated insulin regimen (70% insulin degludec/30% insulin aspart) at 32 units daily in combination with a sulfonylurea, selected due to concerns regarding adherence to more complex regimens. He was subsequently transferred to an inpatient psychiatric unit for the management of a relapse of schizophrenia. Over the following two months, strict glycaemic control was maintained with insulin administered by a community nursing service.

On presentation, he appeared cachectic and pale and was unable to stand due to syncope. Vital signs are summarised in Table 1, with marked orthostatic hypotension noted. Neurological examination revealed preserved sensation to light touch and globally intact muscle strength. Visual acuity appeared reduced, with difficulty reading staff name badges. Notably, HbA1c had fallen to 6.7% (50 mmol/mol), representing a reduction of 9.4 percentage points over three months. Laboratory investigations are summarised in Table 2. Haemoglobin level was mildly reduced. Inflammatory markers, cardiac troponin, and renal and liver function tests were within reference ranges. He was resuscitated with intravenous fluids and commenced on fludrocortisone for orthostatic hypotension and domperidone for suspected gastroparesis. 

**Table 1:** Vital signs

Parameter	Result
Supine systolic blood pressure	120 mmHg
Standing systolic blood pressure	60 mmHg
Heart rate	110 beats per minute
Capillary blood glucose	4.6 mmol/L

**Table 2:** Laboratory investigations

Parameter	Result	Reference range
Glycaemic control		
Glycated haemoglobin (HbA1c)	6.7% (50 mmol/mol)	<7.0% (<53 mmol/mol)
Prior glycated haemoglobin (3 months earlier)	16.1% (151 mmol/mol)	<7.0% (<53 mmol/mol)
Haematology and biochemistry		
Haemoglobin	115 g/L	130–180 g/L
Renal function (eGFR)	>90	>90
Liver function tests	ALP 51 U/L; GGT 16 U/L; ALT 4 U/L; total bilirubin 6 µmol/L	ALP 30–110 U/L; GGT 0–50 U/L; ALT 0–40 U/L; bilirubin 1–20 µmol/L
Inflammatory markers (C-reactive protein)	<5 mg/L	<5 mg/L
Cardiac troponin	2	<26
Endocrine investigations		
Early morning serum cortisol	190 nmol/L	>140 nmol/L
Short Synacthen test	811 nmol/L	Peak cortisol ≥500 nmol/L
Aldosterone-to-renin ratio	20.2 pmol/mU	<70 pmol/mU
Neuropathy screen		
Vitamin B6 (pyridoxine)	Within reference range	20–125 nmol/L
Vitamin B12 (cobalamin)	323 pmol/L	140–700 pmol/L
Thyroid-stimulating hormone	1.39 mIU/L	0.4–4.0 mIU/L
Serum protein electrophoresis	No monoclonal band	Not applicable
Syphilis serology	Non-reactive	Not applicable
Human immunodeficiency virus serology	Negative	Not applicable
Angiotensin-converting enzyme	20 U/L	20–70 U/L

Computed tomography of the chest, abdomen, and pelvis demonstrated no evidence of malignancy or gastric obstruction (Figure 1). Transthoracic echocardiography showed normal ventricular function with no valvular abnormalities (Figure 2). A gastric emptying study revealed a markedly delayed half-emptying time of 149 minutes (reference range 90 ± 15 minutes). As shown in Table 3, quantitative sudomotor axon reflex testing (QSART) demonstrated globally reduced sweat output with prolonged distal response latencies. The more pronounced distal abnormalities supported postganglionic sympathetic sudomotor dysfunction with a length-dependent pattern of small-fibre (C-fibre) involvement, consistent with autonomic small fibre neuropathy. Nerve conduction studies demonstrated a length-dependent sensorimotor polyneuropathy with both axonal large and small fibre involvement, with the large fibre abnormalities of mild-to-moderate severity (Tables 4, 5).

**Figure 1:**
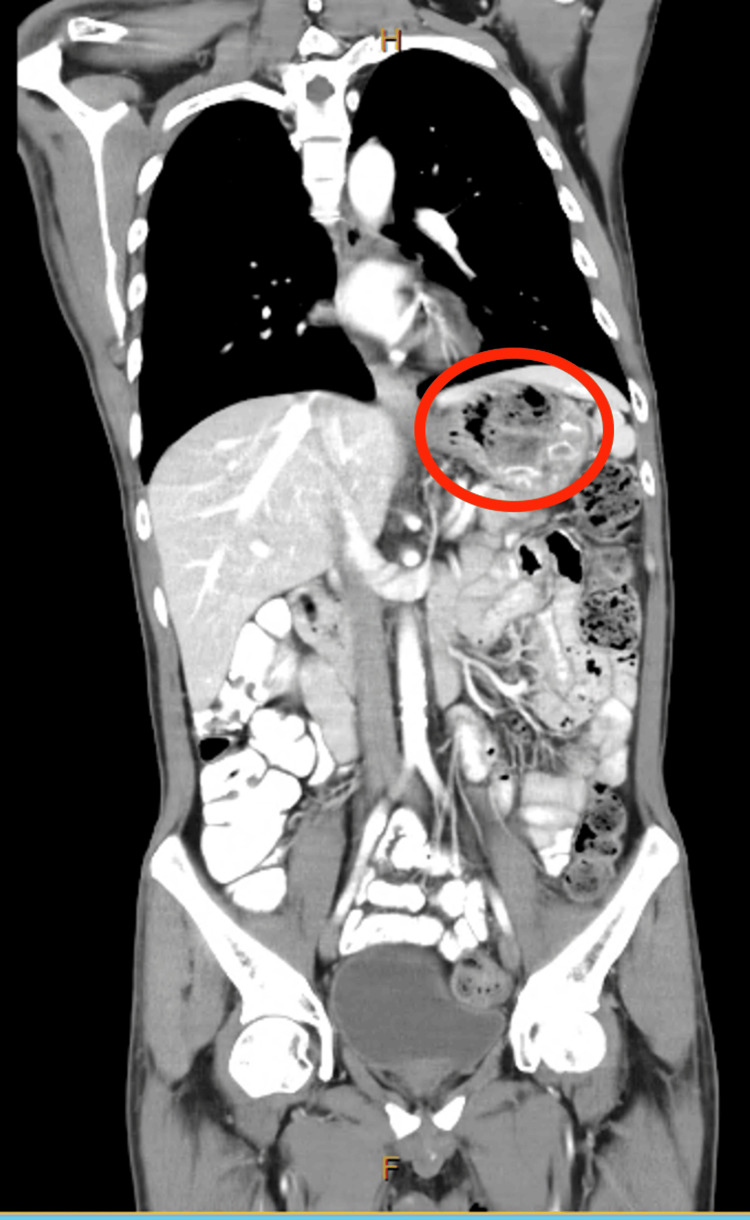
Computed tomography of the chest, abdomen, and pelvis The computed tomography of the chest, abdomen, and pelvis demonstrates a normal gastric appearance without gastric distension, gastric outlet obstruction, or intra-abdominal malignancy, supporting a functional rather than mechanical cause of gastroparesis. The red circle highlights the stomach.

**Figure 2:**
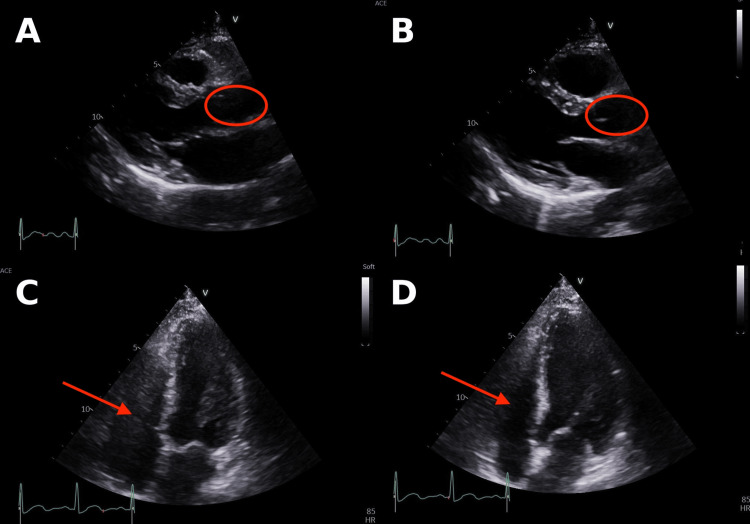
Transthoracic echocardiogram Transthoracic echocardiogram. (A, B) Parasternal long-axis views obtained during end-systole and end-diastole demonstrating a normal left ventricular outflow tract without obstruction (red circles). (C, D) Apical four-chamber views demonstrating normal left ventricular size and preserved systolic function (red arrows), with no structural cardiac cause identified for syncope or hypotension.

**Table 3:** Quantitative sudomotor axon reflex testing Reference ranges are the performing laboratory’s sex-specific (male) normative values.

Test site	Sweat volume (µL)	Volume normal range (µL)	Response latency	Latency normal range (s)	Interpretation
Forearm	0.096	0.5-3.7	4:15 (255 s)	28-191	Reduced volume; prolonged latency
Proximal leg	0.138	0.4-3.9	2:15 (135 s)	47-197	Reduced volume; latency normal
Distal leg	0.07	0.3-3.3	5:00 (300 s)	24-190	Reduced volume; prolonged latency
Foot	0.058	0.2-2.6	5:00 (300 s)	45-190	Reduced volume; prolonged latency

**Table 4:** Motor nerve conduction studies 🔴  Low amplitude/conduction velocity;  NA = not applicable; NR = not recorded

Nerve	Site	Latency (ms)	Amplitude (mV)	Conduction Velocity (m/s)
Right median motor	Wrist-abductor pollicis brevis	3.44	5.3	NA
	Elbow-wrist	8.54	5.1	45.1 🔴
Left peroneal motor	Ankle-extensor digitorum brevis	NR 🔴	NR 🔴	NA
Right peroneal motor	Ankle-extensor digitorum brevis	4.31	0.77 🔴	NA
	Below knee-ankle	14.9	0.47	29.3
	Fibular head-below knee	16.8	0.5	31.6
Left deep peroneal motor	Below knee-tibialis anterior	3.8	3.3	NA
	Above knee-below knee	6.56	2.2	21.7
Left tibial motor	Ankle-abductor hallucis	3.62	3.2 🔴	NA
	Knee-ankle	16.4	2.1	31.3
Right tibial motor	Ankle-abductor hallucis	3.98	4.4	NA
	Knee-ankle	16.6	3.2	29.3
Right ulnar motor	Wrist-abductor digiti minimi	2.6	7.1	NA
	Below elbow-wrist	7.94	6.7	43.1 🔴
	Above elbow-below elbow	9.46	5.4 🔴	65.8
Summary	🔴 Abnormal motor nerves: 5/7			

**Table 5:** Sensory nerve conduction tudies 🔴  Low amplitude / conduction velocity; NA = not applicable; NR = not recorded

Nerve	Site	Peak latency (ms)	Amplitude (µV)	Conduction velocity (m/s)
Right median sensory	Digit II-wrist	3.47 🔴	14.8	50.6
Right median sensory	Palm-wrist	1.98	38.4	61.5
Left superficial peroneal sensory	Calf-medial dorsal cutaneous nerve	NR 🔴	NR 🔴	NR
Right superficial peroneal sensory	Calf-medial dorsal cutaneous nerve	4.81 🔴	3.1	31.7
Left sural sensory	Mid lower leg-lateral malleolus	4.33	2.8	36.9
Right sural sensory	Mid lower leg-lateral malleolus	4.69 🔴	0.91	34.6
Right ulnar sensory	Digit V-wrist	2.97	16.8	52.6
Right ulnar sensory	Palm-wrist	2.27 🔴	28	51.9
Summary	🔴 Abnormal sensory nerves: 5/6			

Despite fluid resuscitation and fludrocortisone, severe orthostatic hypotension persisted. Midodrine (5 mg three times daily) was initiated, followed by caffeine (100 mg three times daily) as an experimental third-line therapy for treatment-resistant orthostatic hypotension. Partial symptomatic improvement was observed following initiation of caffeine therapy, allowing for safe discharge home. However, the relationship between caffeine initiation and clinical improvement remains uncertain, given the concurrent use of other treatments. Neuropathic pain was partially responsive to pregabalin. Ongoing maintenance of stable glycaemic control was advised. The chronology of glycaemic control, symptom onset, investigations, and treatment is summarised in Figure 3.

**Figure 3:**
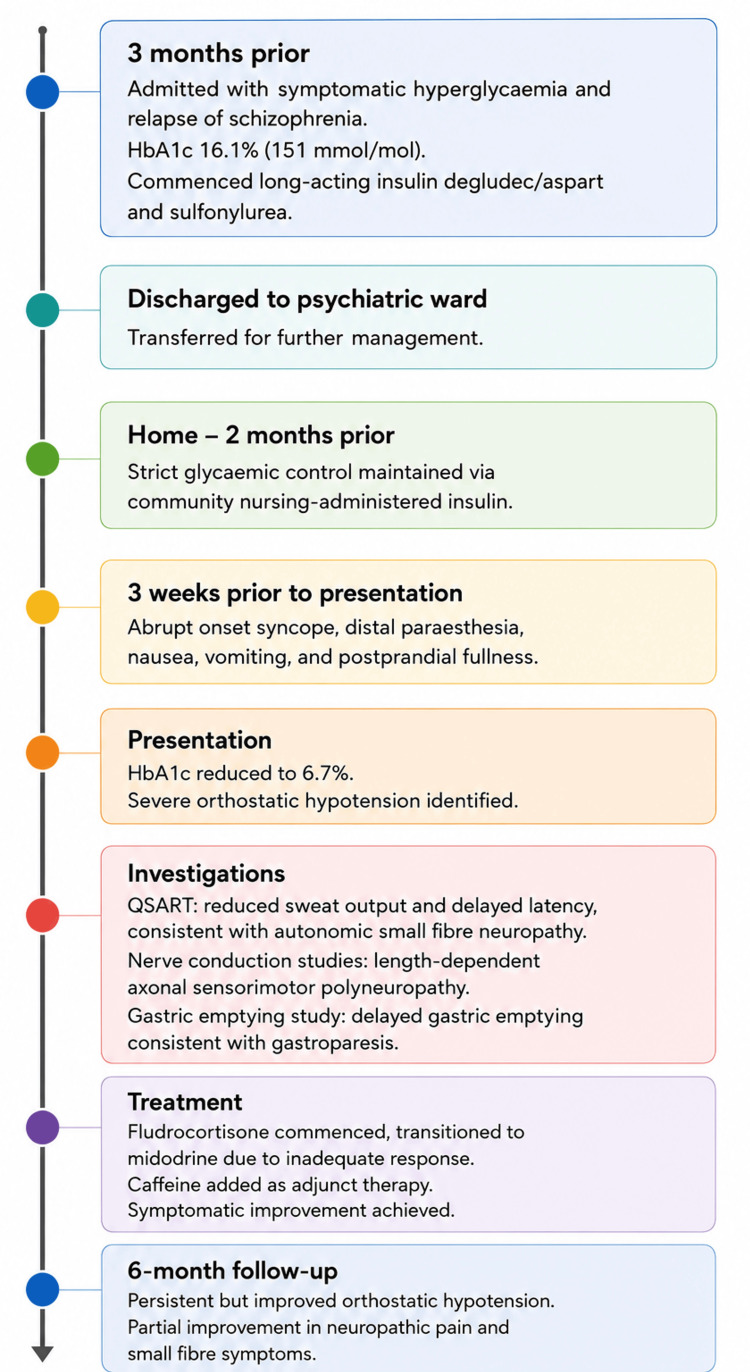
Timeline of symptom onset, investigations, and treatment course HbA1c: glycated haemoglobin; QSART: Quantitative Sudomotor Axon Reflex Test

## Discussion

Gibbons and Freeman (2015) proposed diagnostic criteria for TIND [2]. These criteria include 1) a decrease in HbA1c of ≥2 percentage points over three months, 2) the acute onset of neuropathic pain and/or autonomic dysfunction developing over two weeks and of sufficient severity to prompt medical attention, and 3) symptom onset occurring within eight weeks of the documented HbA1c reduction. Our case meets these criteria, with the patient developing acute autonomic and peripheral neuropathic symptoms following a 9.4 percentage-point reduction in HbA1c over three months. Alternative causes of autonomic neuropathy were also considered, including nutritional, infectious, paraproteinaemic, endocrine, and infiltrative neuropathies, which are recognised causes of small fibre and autonomic neuropathy [6]. In our case, vitamin B12 and thyroid-stimulating hormone levels were within reference ranges, HIV and syphilis serology were negative, serum protein electrophoresis demonstrated no monoclonal band, and angiotensin-converting enzyme levels were normal, making these alternative aetiologies less likely. Diabetic neuropathic cachexia (DNC) is another important differential diagnosis to consider, characterised by the triad of mood disturbance, profound weight loss, and transient, painful, bilateral length-dependent peripheral neuropathy [7]. In this case, the onset of autonomic and peripheral neuropathy following a rapid reduction in HbA1c makes TIND the primary diagnosis. However, concurrent cachexia secondary to gastroparesis, together with mild anaemia (haemoglobin 115 g/L), may also have contributed to the severity of the presentation.

The prevalence of TIND has been reported to be as high as 10.9% in a study of 954 patients across four years in a tertiary referral diabetic neuropathy clinic [2]. The exact pathophysiology of TIND remains incompletely understood. One theory is that the microcirculatory changes seen in chronic hyperglycaemia, namely, arteriovenous shunting, are unable to remodel at the same rate as the reduction in serum glucose, leading to endoneuronal ischaemia [3]. It has also been suggested that glucose deprivation may lead to direct cellular apoptosis [4]. More recently, it has been proposed that shifts in glucose availability may induce metabolic flux-related oxidative stress, mitochondrial dysfunction, and bioenergetic failure within peripheral nerves [5]. Following acute small-fibre injury, the severe neuropathic pain characteristic of TIND is thought to relate in part to aberrant regeneration and ectopic firing of regenerating small nerve fibres [5]. Identified risk factors for TIND include higher pre-treatment HbA1c, greater magnitude of HbA1c reduction, and a history of weight loss [2,8]. As shown in Table 6, Gibbons and Freeman demonstrated a strong relationship between the magnitude of HbA1c reduction and the absolute risk of developing TIND, reporting an approximately 20% absolute risk with a 2-3% reduction in HbA1c over three months, which increased to greater than 80% when HbA1c reduction exceeded 4% over the same period [2]. Notably, the method by which glycaemic control is achieved, whether via insulin, oral hypoglycaemic agents, or lifestyle modification, does not appear to significantly influence the risk of developing TIND [1]. 

**Table 6:** Risk estimates based on the magnitude of HbA1c reduction over a three-month period. Source: Gibbons and Freeman (2015) [2]

HbA1c reduction over three months (%)	Estimated absolute risk of TIND (%)
2-3	~20
>4	>80

Neuropathic pain and autonomic dysfunction should be evaluated with nerve conduction studies, including small fibre interrogation where available. A recent report published by Schueller et al. (2025) described a case of TIND with orthostatic hypotension following a rapid HbA1c reduction from 15.0% to 7.1% over five months [9]. Autonomic dysfunction in that report was inferred clinically from orthostatic hypotension alone, without objective autonomic small fibre testing. By contrast, our case provides objective confirmation of autonomic neuropathy through QSART, demonstrating globally reduced sweat output and prolonged response latencies with a length-dependent distribution, consistent with postganglionic sympathetic C-fibre dysfunction. This represents a clinically important distinction, as objective autonomic testing strengthens diagnostic certainty and further characterises the autonomic manifestations of TIND, which remain relatively under-reported in the literature.

Orthostatic hypotension is defined as a sustained reduction in systolic blood pressure of ≥20 mmHg or diastolic blood pressure of ≥10 mmHg within three minutes of standing [10]. Additional autonomic investigations, including tilt-table testing, autonomic reflex testing, and quantitative sudomotor axon reflex testing (QSART), may further support a neurogenic aetiology [10]. In our case, the marked orthostatic systolic blood pressure reduction from 120 mmHg supine to 60 mmHg standing, together with abnormal QSART findings demonstrating postganglionic sudomotor dysfunction, supported the diagnosis of neurogenic orthostatic hypotension secondary to autonomic neuropathy.

Management of peripheral and autonomic neuropathy is largely supportive. Neuropathic pain is often treated with antiseizure medication or antidepressants [11]. Gastroparesis should be confirmed via a gastric emptying study, with management focused on dietary modifications and the use of prokinetic agents [12]. Non-pharmacological strategies for orthostatic hypotension include increased salt and fluid intake, head-of-bed elevation during sleep, slow positional changes, and the use of abdominal binders or waist-high compression stockings [13]. Pharmacological options aim to expand intravascular volume with fludrocortisone or increase peripheral vascular resistance using agents such as midodrine, droxidopa, or norepinephrine reuptake inhibitors [13]. Caffeine was trialled in our case as an adjunctive third-line therapy for treatment-resistant neurogenic orthostatic hypotension. Caffeine has demonstrated modest benefit in small studies of neurogenic orthostatic hypotension through antagonism of adenosine receptors and augmentation of sympathetic vasoconstriction, although evidence remains limited [14].

The optimal approach to glycaemic management following a diagnosis of TIND remains unclear. Chantelau and Meyer-Schwickerath proposed the idea of "permissive hyperglycaemia" for managing early worsening of diabetic retinopathy from rapid reduction in HbA1c [15]. There is currently no evidence to support permissive hyperglycaemia as a routine treatment strategy for TIND. Gibbons (2017) recruited 26 individuals with type 1 diabetes who had TIND. Of these, 19 maintained stable glycaemic control, defined as having HbA1c ranges of 6-8% (42-64 mmol/mol, respectively) and had a clinical improvement in symptoms of peripheral and autonomic neuropathy over an 18-36-month period. Conversely, individuals whose glycaemic control fluctuated significantly, moving from poor control (HbA1c >10%, >86 mmol/mol) to good control (HbA1c <8%, <64 mmol/mol), experienced a worsening of symptoms [16]. More recent paediatric and adolescent case series have highlighted the heterogeneous clinical course of TIND and demonstrated that autonomic complications, including gastroparesis, may persist despite subsequent stabilisation of glycaemic control [8]. These findings suggest that once TIND develops, maintaining stable glycaemic control is critical, whereas ongoing fluctuations or rapid correction after periods of hyperglycaemia are associated with poorer outcomes [16]. 

It has been proposed that restricting an improvement in HbA1c to no more than two percentage points over three months should reduce the likelihood of developing TIND [1,2]. However, this strategy remains theoretical, as no prospective studies have validated its efficacy. Furthermore, implementing a gradual reduction in HbA1c can be challenging in routine clinical practice. In our patient, schizophrenia and limited engagement with healthcare services increased the risk of loss to follow-up, making gradual insulin titration less feasible. Continuous glucose monitoring may facilitate safer and more gradual glucose lowering by identifying rapid glycaemic shifts and enabling closer insulin titration. Such strategies may help reduce the risk of TIND in high-risk patients.

## Conclusions

In summary, this case highlights the importance of recognising TIND as a differential diagnosis in patients presenting with syncope and postural hypotension with a history of rapid glycaemic correction. Although this phenomenon has been recognised in the literature, it is relatively under-recognised compared to peripheral neuropathy and can cause significant morbidity. Preventing TIND is based on implementing a graded reduction in HbA1c; however, an individualised approach should always be taken by clinicians, balancing a patient’s risk of developing TIND against their risk of developing long-term complications of hyperglycaemia. Further research is warranted to validate the efficacy of graded HbA1c reduction in preventing TIND and to provide clinicians with evidence-based guidance when discharging patients with permissive hyperglycaemia.
